# Methodological considerations in comparing left bundle branch pacing and right ventricular pacing for atrial fibrillation outcomes

**DOI:** 10.1016/j.hroo.2026.06.029

**Published:** 2026-07-16

**Authors:** Muhammad Fayyaz Riaz, Suhail Ahmed, Hayat Asghar

**Affiliations:** Liaquat University of Medical & Health Sciences (LUMHS), Jamshoro, Pakistan

We read with great interest the study by Ramos-Maqueda et al,^1^ reporting a reduced incidence of device-detected atrial fibrillation (AF) among patients undergoing left bundle branch pacing (LBBP) compared with right ventricular pacing. The authors should be commended for conducting a prospective 2-center study addressing an important clinical question. As physiologic pacing strategies continue to gain attention, their findings provide valuable evidence supporting the potential role of LBBP in reducing AF burden among patients requiring ventricular pacing.

The authors rightly acknowledge the non-randomized design as a limitation, but several methodological points deserve additional attention. Allocation by operator preference can introduce selection bias. Further, LBBP procedures were done by 4 experienced operators, whereas right ventricular pacing implantations involved different clinicians, which may well have influenced success rates and outcomes.^2^ Blinding was absent during manual review of AF episodes, which risks verification bias, especially for brief runs.^3^ The clearest signal of benefit appeared among patients with a ventricular pacing burden above 20%, yet this cutoff was not prespecified and looks data-driven, so the observation should be seen as hypothesis-generating, not confirmatory.^4^

Finally, although inclusion of patients with preserved systolic function enhances the study’s applicability, the specific contribution of true left bundle capture remains uncertain.^5^ Additional adequately powered randomized studies are warranted to clarify these observations and further define the role of LBBP in AF prevention.
